# Case Report: Endoscopic radiofrequency ablation with radial-EBUS and ROSE

**DOI:** 10.3389/fmedt.2023.1022220

**Published:** 2023-01-20

**Authors:** Paul Zarogoulidis, Wolfgang Hohenforst-Schmidt, Vasileios Papadopoulos, Eleni-Isidora Perdikouri, Nikolaos Courcoutsakis, Konstantinos Porpodis, Dimitrios Matthaios, Kostas Trigonakis

**Affiliations:** ^1^ Pulmonary Department, General Clinic Euromedica, Thessaloniki, Greece; ^2^ 3rd Surgery Department, AHEPA University Hospital, Aristotle University of Thessaloniki, Thessaloniki, Greece; ^3^ Sana Clinic Group Franken, Department of Cardiology/Pulmonology/Intensive Care/Nephrology, “Hof” Clinics, University of Erlangen, Hof, Germany; ^4^ Oncology Department, University General Hospital of Larissa, Larissa, Greece; ^5^ Oncology Department, General Hospital of Volos, Volos, Greece; ^6^ Radiology Department, Democritus University of Thrace, Alexandroupolis, Greece; ^7^ Pulmonary Department, `G. Papanikolaoù` General Hospital, Aristotle University of Thessaloniki, Thessaloniki, Greece; ^8^ Oncology Department, General Hospital of Rhodes, Rhodes, Greece; ^9^ Vascular Surgery Department, General Clinic Euromedica, Thessaloniki, Greece

**Keywords:** lung cancer, ablation, radial-EBUS, ROSE, navigation, FNA

## Abstract

**Background:**

Single pulmonary nodules are a common issue in everyday clinical practice. Currently, there are navigation systems with radial-endobronchial ultrasound and electromagnetic navigation for obtaining biopsies. Moreover, rapid on-site evaluation can be used for a quick assessment. These small lesions, even when they do not have any clinically significant information with positron emission tomography, are important to investigate.

**Case description:**

Radiofrequency and microwave ablation have been evaluated as local treatment techniques. These techniques can be used as therapy for a patient population that cannot be operated on. Currently, one verified operating system is used for endoscopic radiofrequency ablation through the working channel of a bronchoscope.

**Conclusion:**

In our case, a new system was used to perform radiofrequency ablation with long-term follow-up.

## Introduction

Single pulmonary nodules are a clinical issue that needs special attention. A nodule is defined as a lesion up to 3 cm. Currently, we can use imaging techniques with computed tomography (CT) and positron emission tomography (PET-CT). If a nodule is ≤1 cm, then positron emission tomography will not provide us with useful clinical information unless the uptake of SUV is ≥3. In the case of these very small lesions, we can order a follow-up every 2–3 months, and if they are increased in size, we can proceed to biopsy or surgery. In the case of nodules ≥1.1 cm, positron emission tomography can provide us with useful clinical information based on the SUV uptake; however, again such a lesion might be a cancerous lesion with a low metabolic rate, and we might have a false-negative result (1, 2). However, all these guidelines have not been updated according to the new endoscopic techniques that have been developed in recent years. Nowadays, radial-endobronchial ultrasound (EBUS) and electromagnetic navigation can be used to perform a biopsy in pulmonary nodules with a high rate of efficiency (3–6). Moreover, over the past decade, rapid on-site evaluation (ROSE) has been used to identify malignant lesions (7). In the case of a single nodule without any other cancer metastatic site, surgery or ablation is performed. Currently, ablation is performed under computed tomography guidance with radiofrequency, microwave, and thermosphere probes. In our case, a Covidien radiofrequency system was used.

## Case presentation

**Figure 1:**
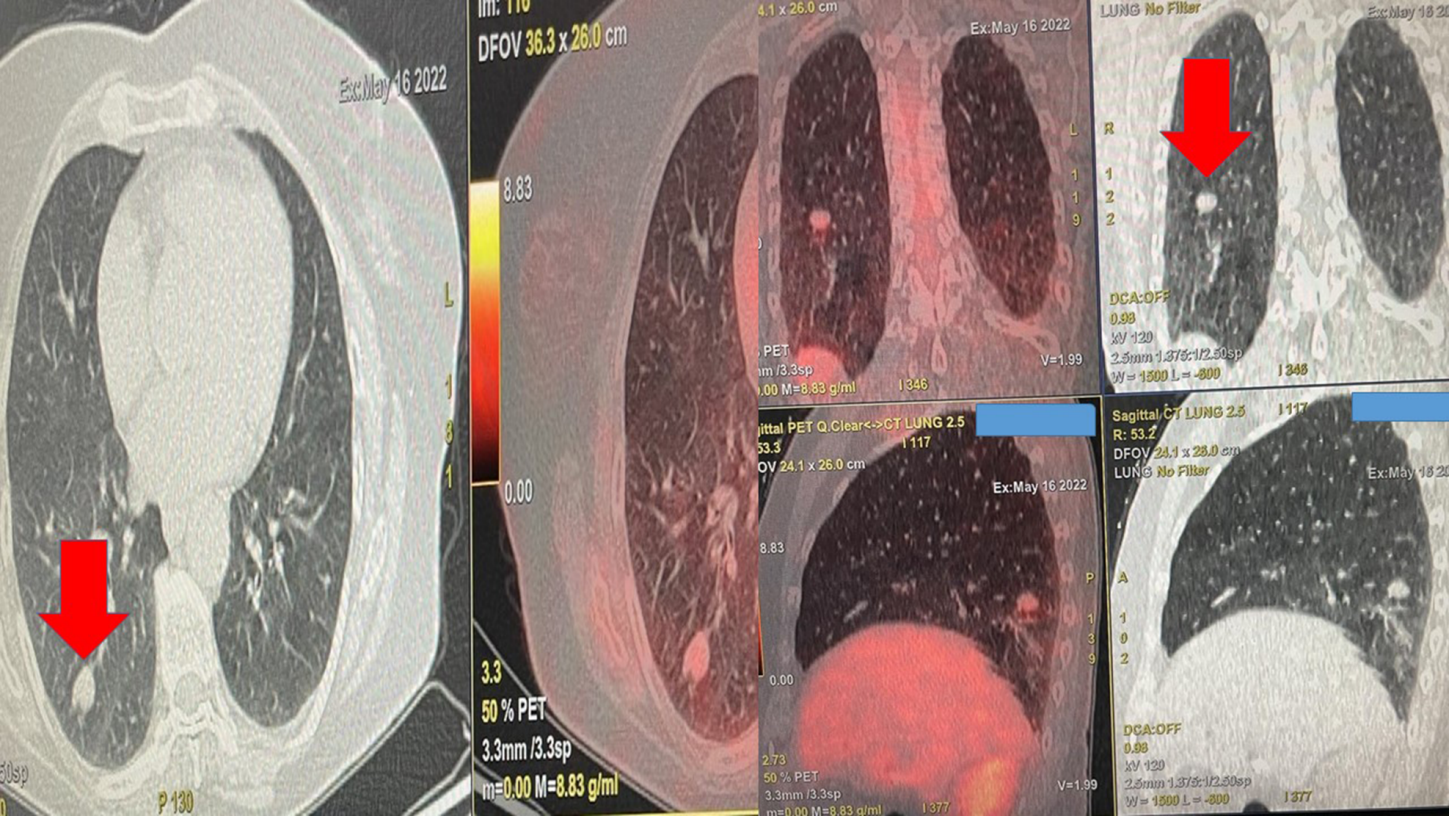
Positron emission tomography findings upon diagnosis. The red arrow indicates the single nodule.

**Figure 2:**
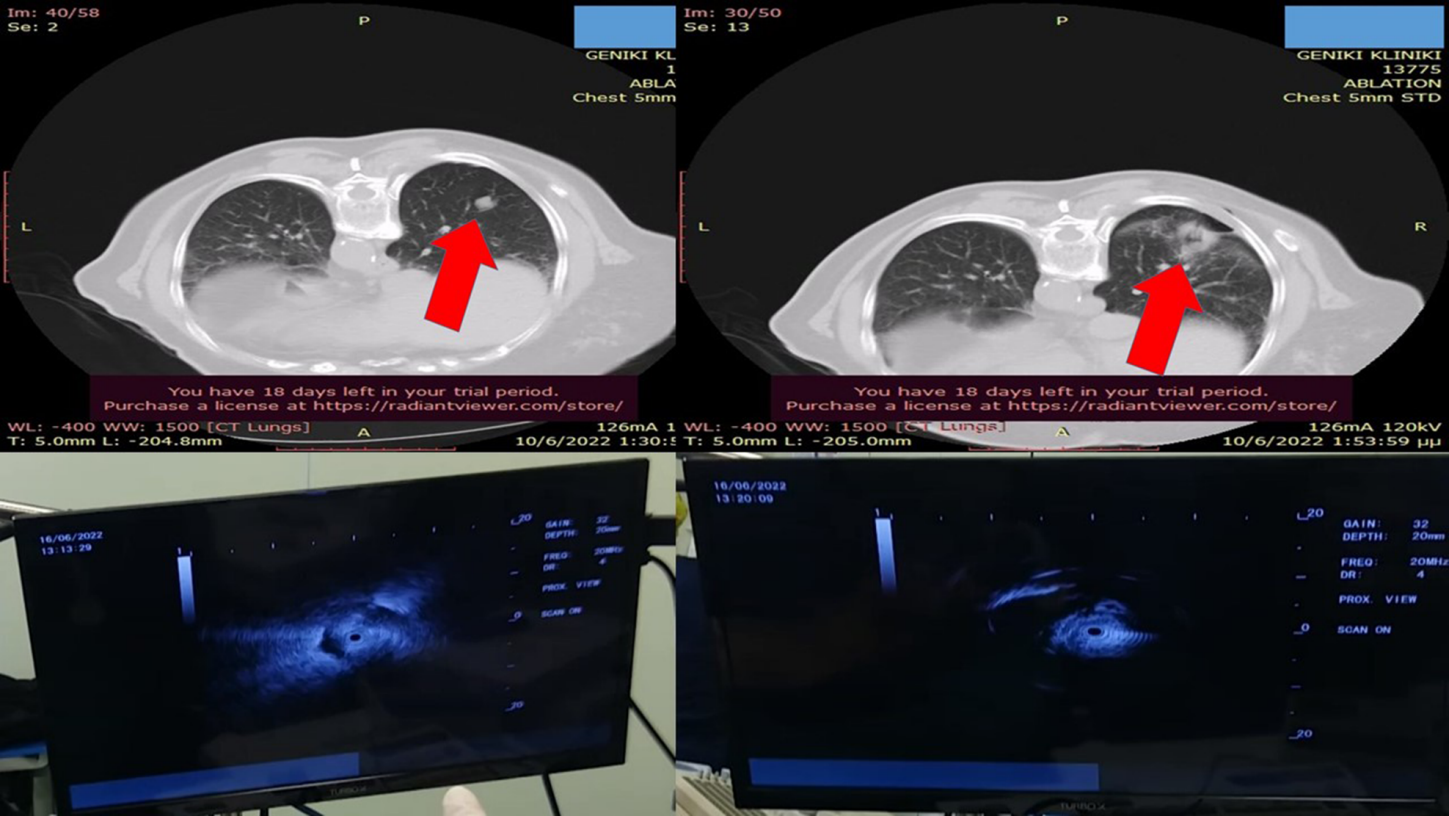
Left upper-row red arrow indicates the lesion before ablation, the left lower row demonstrates the lesion sign with radial-EBUS, the right upper-row red arrow indicates the area after two sessions of thermal effect with radiofrequency ablation, and the right lower row demonstrates the lesion sign after the ablation. EBUS, endobronchial ultrasound.

A 65-year-old woman, nonsmoker, was diagnosed in our outpatient cabinet with a single pulmonary nodule in the left lower lobe. Positron emission tomography was performed, and a significant SUV uptake of 8.9 was observed in the nodule (Figure 1). The patient had a persistent cough for 3 weeks, she did not report fever, and her laboratory findings were within normal range. It was decided to have a radial-endobronchial ultrasound with C-ARM for diagnosis with ROSE and convex-endobronchial ultrasound with rapid on-site evaluation for staging (Figure 2). Indeed, the ROSE technique evaluated non-small-cell lung cancer (NSCLC) for the single pulmonary nodule, and lymph node staging by ROSE revealed no metastasis in the lymph nodes or other organs. Local radiofrequency ablation with an endoscopic radiofrequency probe was proposed.

## Treatment procedure

**Figure 3:**
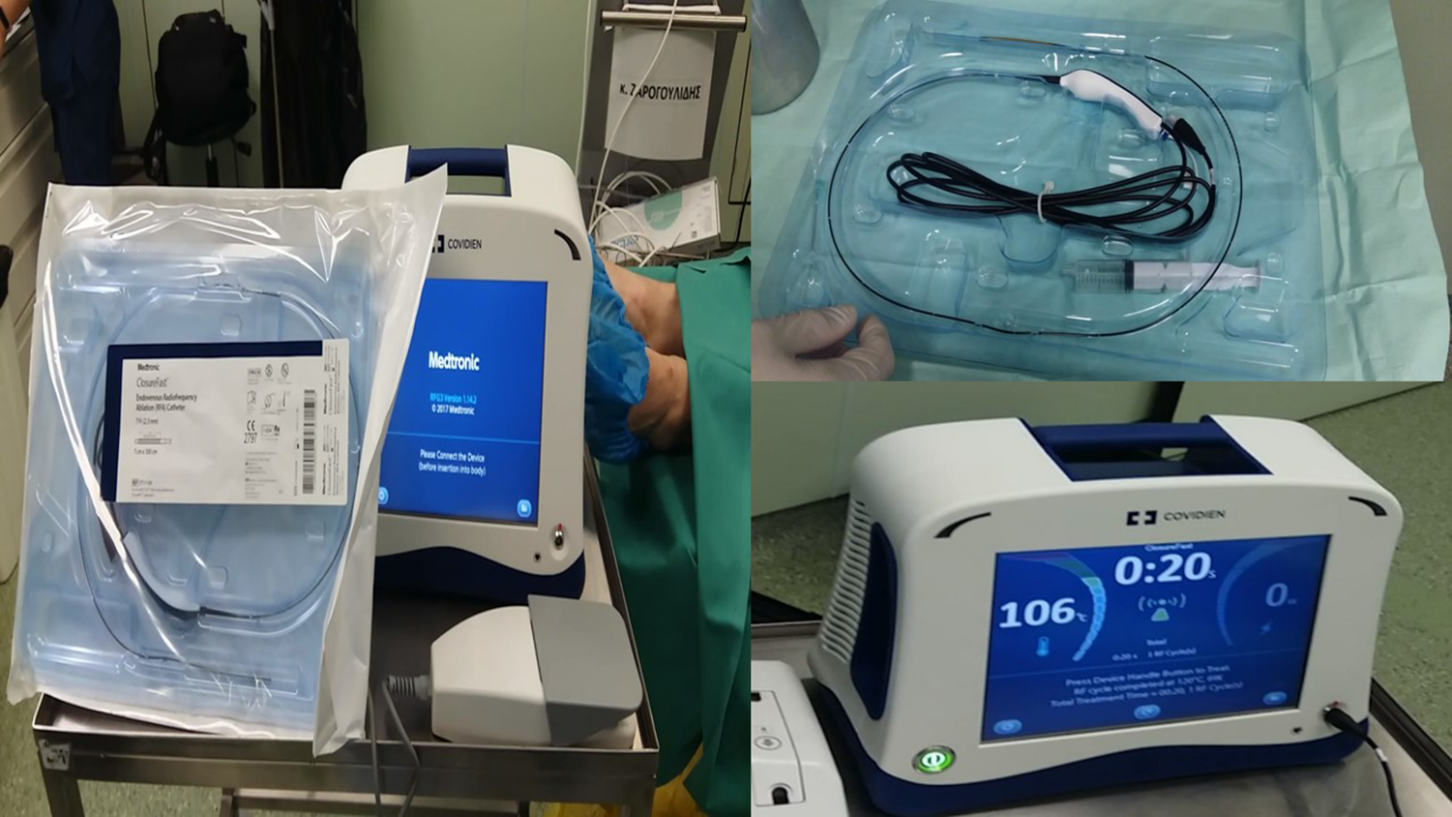
Covidien endovascular radiofrequency catheter. On the left, one can see both the generator and the ablation probe. On the upper right is the probe in magnification, and on the lower right is the Covidien generator.

The patient agreed to the minimally invasive procedure of endoscopic radiofrequency ablation. We used two sessions of 20 s each with 40 W (Figure 3). The patient was intubated with a 7.5-mm tracheal tube with a high-volume, low-pressure cuff and was under jet ventilation. The consistency of the nodule was evaluated before and after the procedure with radial-endobronchial ultrasound, and computed tomography verified the results. The patient had a spirometry and heart examination before the procedure to be prepared in the case of lobectomy. We had, at our disposal, balloon blockers and hemostatic dry powder in the case of hemorrhage. After 1 year of follow-up, the patient is disease-free. We performed computed tomography scanning with i.v. contrast infusion after 40 days for disease evaluation and PET-CT after 3 months of the procedure according to guidelines.

## Discussion

To perform a biopsy on a single pulmonary nodule, we use computed tomography-guided needles, usually 18G, or radial-endobronchial ultrasound through bronchoscopes. In the second method, we can use biopsy forceps for histology samples, needles for a fine needle biopsy, usually 22G, or brushes for cytology samples (3, 4). In the case of cytology material, rapid on-site evaluation can be performed, and a positive result is enough for diagnosis (8). Electromagnetic navigation is another technique that can be used, and currently, we have two platforms, the Covidien endovascular radiofrequency catheter, formerly Medtronic, and the ARCHIMEDES system from Bronchus (9, 10). The ARCHIMEDES system has the advantage of additionally providing information regarding the surrounding vessels next to the lesion. Monarch is another platform regarded as a robotic-assisted method that we use under the guidance of computed tomography (11). In the case of a single nodule positive for NSCLC without distant metastasis, we can perform surgery or ablation. Regarding surgery, we have two techniques, video-assisted thoracic surgery (VATS) and robotic-assisted thoracic surgery (RATS) (12, 13). However, ablation is another solution for these patients or those who cannot undergo surgery due to comorbidities. Until recently, we performed ablation under computed tomography with radiofrequency or microwave needles. Both probes are equally efficient; however, the ablation time is less with microwave needles. Each probe has its advantages and disadvantages, which will not be discussed here. The main adverse effects are pneumothorax, hemothorax, or bleeding (14). Currently, we have evaluated one system for endoscopic radiofrequency ablation (15). There are technical differences between the percutaneous ablation systems and the endobronchial ultrasound; however, the result is the same. Moreover, the same system that produces steam for emphysema treatment has been used as a thermal endoscopic ablation system in Australia (16). For the first time, a Covidien endovascular radiofrequency system was used with more watts and double time based on the previous knowledge from the radiofrequency probes used for computed tomography ablation. The effect of ablation was evaluated with radial-EBUS. The results were encouraging based on our long-term follow-up, and technical aspects of the procedure will be improved since we have two catheters of short and long lengths, 3 and 7 mm, respectively. Finally, computed tomography scanning with i.v. contrast infusion was used to evaluate disease relapse after 30–40 days of ablation. Positron emission tomography was avoided under several weeks since there is going to be a pseudo-positive result of increased SUV due to local inflammation.

## Conclusion

The most important issue is that we can evaluate the effect of radial-EBUS on site after every session of ablation and decide whether to continue or not. We did not have any adverse effects, and the procedures were terminated after 45 min. The only reason for the delay was the approach of the nodule.

## Data Availability

The raw data supporting the conclusions of this article will be made available by the authors, without undue reservation.
